# Study on performance degradation and damage modes of thin-film photovoltaic cell subjected to particle impact

**DOI:** 10.1038/s41598-020-80879-w

**Published:** 2021-01-12

**Authors:** Kailu Xiao, Xianqian Wu, Xuan Song, Jianhua Yuan, Wenyu Bai, Chenwu Wu, Chenguang Huang

**Affiliations:** 1 Institute of Mechanics, Chinese Academy of Sciences, No.15 Beisihuanxi Road, Haidian District, Beijing, 100190 China; 2 School of Engineering Science, University of Chinese Academy of Sciences, Beijing, 100049 China; 3 College of Electrical Engineering and Renewable Energy, Three Gorges University, Yichang, 443002 China; 4 Department of Medical Engineering, California Institute of Technology, Pasadena, CA 91125 USA

**Keywords:** Energy science and technology, Engineering

## Abstract

It has been a key issue for photovoltaic (PV) cells to survive under mechanical impacts by tiny dust. In this paper, the performance degradation and the damage behavior of PV cells subjected to massive dust impact are investigated using laser-shock driven particle impact experiments and mechanical modeling. The results show that the light-electricity conversion efficiency of the PV cells decreases with increasing the impact velocity and the particles’ number density. It drops from 26.7 to 3.9% with increasing the impact velocity from 40 to 185 m/s and the particles’ number densities from 35 to 150/mm^2^, showing a reduction up to 85.7% when being compared with the intact ones with the light-electricity conversion efficiency of 27.2%. A damage-induced conversion efficiency degradation (DCED) model is developed and validated by experiments, providing an effective method in predicting the performance degradation of PV cells under various dust impact conditions. Moreover, three damage modes, including damaged conducting grid lines, fractured PV cell surfaces, and the bending effects after impact are observed, and the corresponding strength of each mode is quantified by different mechanical theories.

## Introduction

Thin-film multi-junction photovoltaic (PV) cells made from the compounds of III–V materials have been widely adopted due to their high light-electricity conversion efficiency and low areal mass density^1,2^. Among all possible candidates, the monolithic triple-junction cells (GaInP/GaInAs/Ge) are extensively used because of their high resistance to thermal degradation^3^. PV modules are often installed in the harsh outdoor environment and thus tend to suffer several kinds of faults, causing unexpected safety issues, power losses, and even fire hazards^4–6^. Actually, not only will the transport of the PV modules but also their storage and unsuitable handling during installation lead to performance reduction or potential faults^7^. Dust is an unavoidable factor that affects solar PV module performance and is considered as one of the major factors that contribute to the formation of hot spots^8,9^ in a PV module. Dust impact loadings such as windy, sandy deposition, and debris impact, which can significantly reduce the efficiency of PV cells in an intrinsically coupled pattern and even lead to ultimately failure^8–10^, posing great challenges in their application in-field use.

There will be inevitable impact or deposition of dust that can be induced by human activities (i.e. industrial emissions, construction debris, highway activities and so on) or natural environment (i.e. sandy weather, desert storms, volcanic eruptions and so on)^11^. The previous study for the impact of dust mainly focused on the power output and efficiency change. The wind-tunnel experiments^12^ and in-situ observations in real environments are generally designed to realize the impact behavior of particles to evaluate the effects of dusty and sandy environments on ground-based PV panels. Ahmed and Israa^13^ investigated the impact of dust on the performance of a PV system in Sharjah desert, showing significant deterioration of efficiency because of the dust accumulation. A linear relationship between the dust density and the normalized PV power with a drop of 1.7% per g/m_2_ was observed for a period over 5 months. The study by Pavan et al*.*^14^ showed that the efficiency of polycrystalline silicon PV panels decreased by approximately 5% after 12-month exposure to dust pollution. Javed et al*.*^15^ investigated the dust characteristics and accumulated rate over various exposure periods. They concluded that the dust particles tended to agglomerate for a longer exposure time. The results by Chen et al*.*^16^ indicated that the dust reduced the PV output power by 7.4% for one week in East China. Memiche et al*.*^17^ investigated the effects of dust and weather conditions on the PV system performance in a Saharan environment, and it showed that the electrical power loss was about 30% in desert regions. The research conducted by Gholami et al*.*^18^ showed that the dust impact and accumulation caused a 21.47% reduction in the power output for a 70-day experiment in Iran. Alnaser et al*.*^19^ recorded a 10% degradation when the PV modules were exposed to the local outdoor environment for 100 days. Frage et al*.*^20^ investigated the reduction of peak power and the results showed that approximately 13.7% degradation during 23 days in Minas Gerais, Brazil. It is indicated that the effects of dust on the degradation of PV module's performance would waste the natural resources severely.

The micro dust with diameters of sub-millimeter is generally more destructive for PV cells since they are hard to be detected and excluded, causing more irradiance loss. It is, therefore, of great significance to evaluate the effects of mechanical impact from micro-particles on the performance of PV cells in the laboratory to optimize the cell design and enhance its durability with the ultimate goal of reducing the use of natural resources. However, the small scale of a single PV cell puts forward a serious issue to realize the controllable impact tests in the laboratory. It remains challenging to accelerate micro-particles in a laboratory setting as well as impinge them on the PV cells with controllable velocity. Furthermore, compared with the high-speed impact experiments with relatively volumetric cells, in-situ observation of the impact from micro-particles poses additional difficulties in experimentally validating the impact resistance of the single PV cell^21^.

In the present work, a novel acceleration experimental method based on the previous results of Lee et al*.*^22^ and Hassani-Gangaraj et al*.*^23,24^ with repeatable and controllable impact velocity in the laboratory is developed to investigate the effects of the windy and sandy environment on the performance change of a typical type of PV cells. Considering the superposition effect, a scenario of a single impact with a relatively high-velocity of micro-particles is designed to approximately simulate the low-velocity long-term impacts in the real environment. This experimental method can provide the possible performance change of PV modules before the manufacture and installation of integrated PV modules and support the factory to optimize the design of PV cells. Here, the sandy environment is simulated by laser-shock-driven massive particle impact. The light-electricity performance of the damaged PV cells after impact is measured and compared with those of the intact ones. The damage-induced conversion efficiency degradation (DCED) model is also developed to quantitatively capture the effect of dimensionless damage-degree and impact area on the conversion efficiency degradation. The key parameters, such as the particle diameter, the number density of the particles, and the strengths of PV cell materials, which may affect the damage-degree of the PV cell, are taken into account. Besides, the impact-induced damage behavior, which is observed by Optical Microscope (OM), Scanning Electron Microscope (SEM), and Energy-Dispersive X-ray Spectrometer (EDS), is classified into three damage modes. Meanwhile, the related failure mechanisms are discussed based on mechanical models to further evaluate the impact resistance ability of the PV cells.

## Results and discussion

### Impact process of micro-particles

**Figure 1:**
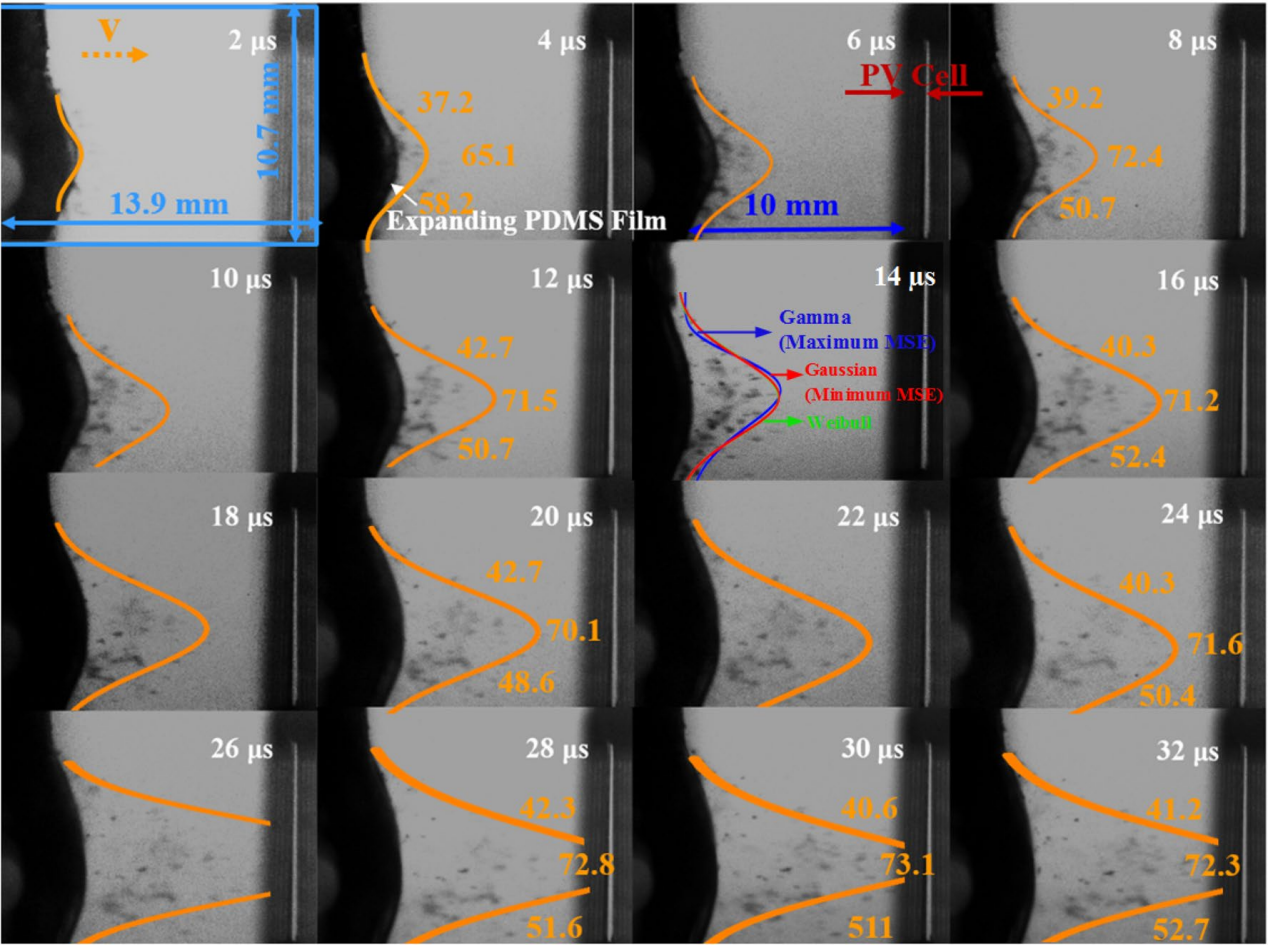
Impact history of particles on the PV cell. It is clearly shown that the radial distribution of velocities of the particles is nearly in Gaussian distribution (The unit for velocities: m/s).

Considering the superposition effect, a scenario of a single impact with relative high-velocity of micro-particles is designed to simulate the low-velocity long-term impacts in-field measurement. The impact velocity, *v*_i_, could be measured from the images captured by the high-speed camera. Figure 1 shows the impact history of a cluster of particles on a PV cell, where the spatial distribution of the particle cluster profiles is depicted by the orange lines, and the average velocities at different sections are measured and marked by the orange data. For this study, PV cells for different particles density are tested. The velocities of particles impinging on the targets vary within the range of 40 ~ 210 m/s. The spatial profile of the laser follows Gaussian distribution, and therefore the velocities along radial direction could be regarded as Gaussian distribution. The Weibull and Gamma distributions are also used to fit the velocity profiles (see Supplementary Fig. S1). It can be seen that the Gaussian distribution gives the best fitting results with the smallest mean square error (MSE). The massive particles with number densities varying from low to high are undertaken in the experiments to roughly represent the uncertainty of mass densities of dust in field measurements. In the present study, the average particle number densities of about *N*_1_ = 35/mm^2^, *N*_2_ = 80/mm^2^, and *N*_3_ = 150/mm^2^ are employed by considering the diversity of the environment.

**Figure 2:**
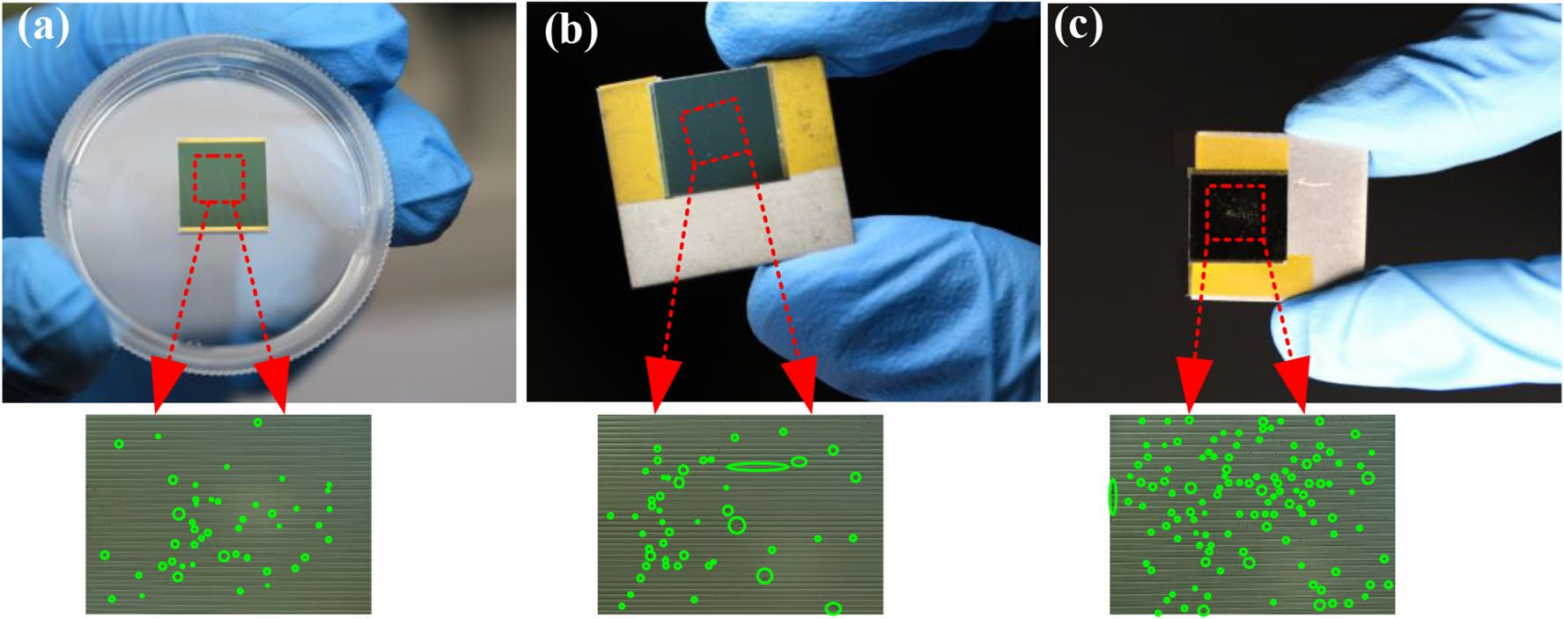
PV cells after impacted by particles with number densities of (**a**) *N*_1_ = 35/mm^2^, *v*_i_ = 65 m/s, (**b**) *N*_2_ = 80/mm^2^, *v*_i_ = 122 m/s and (**c**) *N*_3_ = 150/mm^2^, *v*_i_ = 158 m/s. The major damaged sites in the local enlarged figures were marked by green circles.

Figure 2a–c show the typical macroscopic morphologies and damaged region distribution of the PV cells at *N*_1_ = 35/mm^2^ and *v*_1_ = 65 ± 10 m/s, *N*_2_ = 80/mm^2^ and *v*_2_ = 122 ± 13 m/s, and *N*_3_ = 150/mm^2^ and *v*_3_ = 158 ± 8 m/s after being impacted, respectively. The enlarged impact damage region is marked by the green circle. It is clearly shown that the damage density and the damaged area increase with increasing the particles number density and the impact velocity.

### Light-electricity conversion performance

**Figure 3:**
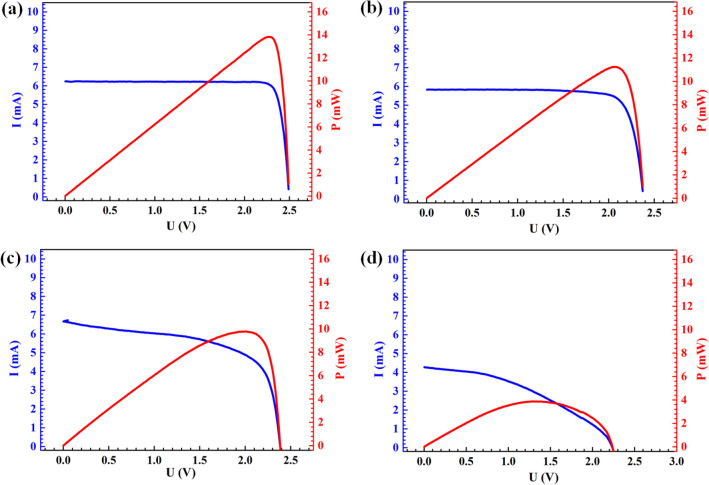
Electrical outputs of (**a**) an intact PV cell, and damaged PV cells impacted by massive particles with (**b**) *N*_1_ = 35/mm^2^, *v*_i_ = 100 m/s (i.e. corresponding to No.3 in Table 1), (**c**) *N*_2_ = 80/mm^2^, *v*_i_ = 135 m/s (i.e. corresponding to No.6 in Table 1), (**d**) *N*_3_ = 150/mm^2^, *v*_i_ = 150 m/s (i.e. corresponding to No.8 in Table 1). The I-V curves are tested by Keithley 2400 source meter under the illumination of AM 1.5, 100 mW/cm^2^ by an Ivtest Station 4000AAA solar simulator.

Damage-degree would affect the light-electricity conversion efficiency during usage. The typical electrical output curves for the PV cells before and after impact are shown in Fig. 3, and the corresponding light-electricity conversion performance degradation in our experiments are listed in Table 1. Figure 3a shows the I–V curve and output electrical power of the intact PV cell for reference. Generally, the accuracy of the I–V test is a little inferior to the external quantum efficiency (EQE)^25^ and SolTrace tools^26^ in experiments. However, it is easier to implement in experiments and the cost is much lower when compared to the other methods, and the accuracy of the I–V test is generally acceptable in engineering. The open-circuit voltage of the PV cell decreases from 4 to 10% and the peak output power decreases from 17 to 73% with increasing impact velocity from 100 to 150 m/s compared to the intact ones. The tendency of short-circuit current dramatically decreases with the increase of the impact velocity and the number density of the particles as shown in Fig. 3c,d. As shown in Table 1, the light-conversion efficiency decreases from 27.1 to 3.9% with increasing the impact velocity from 40 to 185 m/s and the particles’ number densities from 35 to 150/mm^2^, showing a reduction up to 86% when compared to the intact one with the light-electricity conversion efficiency of 27.2%. The complete failure occurs when the velocity exceeds 185 m/s.

**Table 1:** Light-electricity conversion performance of the PV cells before and after impact.

Test no.	Particles number density	Impact velocities	Short-circuit current change	Output electrical power change	Conversion efficiency	Crack morphologies
No.1	0/mm^2^	0 m/s	0	0	27.2%	Intact
No.2 Figure 7(b)	35/mm^2^	40 m/s	− 1%	− 0.4%	27.1%	Damaged grid line
No.3 Figure 7(b)	35/mm^2^	100 m/s	− 6.7%	− 1.8%	26.7%	Damaged grid line
No.4	80/mm^2^	100 m/s	− 7.2%	− 6.3%	25.5%	Damaged grid line
No.5	35/mm^2^	135 m/s	Increase slightly	− 21%	21.5%	Damaged grid line
No.6	80/mm^2^	135 m/s	Increase slightly	− 36.8%	17.2%	Partly peeled-off
No.7	150/mm^2^	135 m/s	Decrease sharply	− 43.8%	15.3%	Partly peeled-off
No.8	150/mm^2^	150 m/s	− 32%	− 77.6%	6.1%	Obvious cracks
No.9	80/mm^2^	185 m/s	− 69%	− 85.7%	3.9%	Obvious cracks
No.10	80/mm^2^	204 m/s	–	–	0	Totally damage
No.11	150/mm^2^	210 m/s	–	–	0	Totally damage

It should be pointed out that the conversion efficiencies do not drastically decline until the impact velocity exceeds 100 m/s for the particles’ number density 35/mm^2^ (as shown in No.2, No.3, and No.4), indicating a threshold impact velocity for light-electricity performance degradation in such a single impact that will be illustrated in next section. The crack morphologies of the damaged PV cells are simply illustrated in Table 1, including the damaged grid line, the partly peeled-off PV cell materials, some apparent cracks in the PV cell, and total fragmentation. The damage behavior affects the performance of PV cells in field usage. Obviously, more severe degradation of electricity performance will be triggered with higher impact velocity and number densities of particles. The degradation of conversion efficiency is also supportive to understand the role of the mechanical impact. Such degradation will cause a dramatic drop in efficiency, leading to huge economic loss.

One phenomenon is that we found a 32% drop of short circuit current and 77.6% reduction of output power as shown in Fig. 3d and No. 8 in Table 1 when *N* = 150/mm^2^, *v*_i_ = 150 m/s. This discrepancy is probably due to that particles impact contributes to the destruction of the P–N junction inside the PV cells, leading to the decrease of conversion capability of impacted units. The open-circuit voltage, the peak output power, and the conversion efficiency degrade with the increase of impact velocity. According to the previous study, the output power reduction could reach about 2.5% ~ 3.4% after two weeks, 3% ~ 4% after three weeks, and 34% ~ 43% after six months for monocrystalline, polycrystalline, and amorphous silicon panels under the outdoor exposure environment^27^. The laser-shock-driven massive high-velocity short-term particles experiment in the laboratory can somewhat reflect the reduction of output power in field measurements for a different period. The scaling law of the damage behavior between the short-time high-velocity micro-particles impact in the laboratory and the long-term low-velocity dust impact in field test might be established based on the total impact kinetic energies, which will help evaluate the performance degradation of PV cells in a practical environment and optimizing the structure design of PV modules in the laboratory.

### DCED model

**Figure 4:**
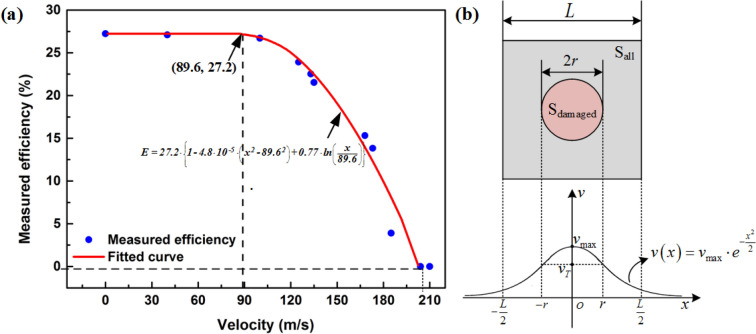
(**a**) Efficiency reduction versus impact velocity of massive particles. (**b**) Schematic of the evaluation model for predicting impact-induced damage of the PV cells by massive particles.

To quantify the effects of impact parameters on the conversion performance of the PV cells, a damage-induced conversion efficiency degradation (DCED) model is developed. The relationship between impact-induced degradation of light-electricity conversion efficiency versus peak impact velocity is shown in Fig. 4a. The conversion efficiency of the intact PV cell is about 27.2 ± 0.3% as indicated in Fig. 4a, which can be regarded as the case with zero impact velocity. With the increasing impact velocity from 135 to 204 m/s under the same number density *N*_2_ = 80/mm^2^ corresponding to Fig. 3c, the light-electricity conversion efficiency declines from 17.2 to 0%. The degradation of conversion efficiency after impact can be estimated by considering the non-linear relationship between the damaged area and the conversion efficiency of PV cells as depicted in Fig. 4a. The density, number density, and diameter of particles in the damaged area as well as the impact velocity are taken into account. The conversion efficiency of the PV cell after impact can be regarded as the summation of contributions from both the intact and the impact regions as follows,

1 $$\begin{aligned} E_{{{\text{PV}}}} & = E_{{{\text{PV}}_{{0}} }} \cdot \left( {1 - \left( {S_{{{\text{damaged}}}} /S_{{{\text{all}}}} } \right)} \right) + E_{{{\text{PV}}_{{1}} }} \cdot \left( {S_{{{\text{damaged}}}} /S_{{{\text{all}}}} } \right) \\ & = E_{{{\text{PV}}_{{0}} }} \cdot \left( {1 - \left( {S_{{{\text{damaged}}}} /S_{{{\text{all}}}} } \right)} \right) + E_{{{\text{PV}}_{{0}} }} \cdot \left( {1 - D} \right) \cdot \left( {S_{{{\text{damaged}}}} /S_{{{\text{all}}}} } \right) \\ & = E_{{{\text{PV}}_{{0}} }} \cdot \left( {1 - D \cdot S_{{{\text{damaged}}}} /S_{{{\text{all}}}} } \right), \\ \end{aligned}$$

where $E_{{{\text{PV}}_{0} }}$ is the efficiency of the intact PV cell, $E_{{{\text{PV}}}}$ denotes the total efficiency of the PV cell after impact, and $E_{{{\text{PV}}_{1} }}$ represents the efficiency of the damaged area of the PV cell. The total area of the PV cell $S_{{{\text{all}}}} = L \times W = 81\,{\text{ mm}}^{2}$, $L = 9\,{\text{ mm}}$ and $W = 9\,{\text{ mm}}$ are the in-plane dimension of the PV cell, $S_{{{\text{damaged}}}}$ stands for the total damaged area, $D$ denotes a dimensionless parameter^28^ with a value in a range of 0–1 representing the average damaged degree of the impacted area. The dimensionless damage-degree of the impact region is dependent on the impact area (area density of particles) and the overloading condition in the impact area (ratio between the impact-induced hydrodynamic pressure and the ultimate strength of the PV cell) and construct as follows,

2 $$D = \alpha \cdot \left[ {\left( {N \cdot \pi \left( {d/2} \right)^{2} } \right)/S_{{{\text{damaged}}}} } \right] \cdot \left\{ {\left[ {\iint\limits_{{S_{{{\text{damaged}}}} }} {\rho_{{\text{P}}} \left( {v^{2} - v_{{\text{T}}}^{2} } \right)/\sigma_{{\text{s}}} dS}} \right]/S_{{{\text{damaged}}}} } \right\},$$

where $N$ is particle number, $d$ denotes average particle diameter, and $v$ represents particle’s impact velocity in the damaged area $S_{{{\text{damaged}}}}$. $\rho_{{\text{P}}}$ is the density of particles, $\sigma_{{\text{s}}}$ is the strength of the PV cell material, $\alpha$ denotes a dimensionless constant, $v_{{\text{T}}}$ denotes threshold velocity for the PV cell to generate damage. In Eq. (2), $\eta$ = $\left( {N \cdot \pi \left( {d/2} \right)^{2} } \right)/S_{{{\text{damaged}}}}$ represents the area density of the massive micro-particles in the damaged area and $\left[ {\rho_{{\text{p}}} \left( {v^{2} - v_{{\text{T}}}^{2} } \right)/\sigma_{{\text{s}}} } \right]$ represents the ratio between the impact-induced hydrodynamic pressure and the ultimate strength of the PV cell, implying the damage-degree of the PV cell. It is to be noted that there should be a lower limit of impact velocity $v_{{{\text{limit}}}}$, for the PV cell to fail completely, i.e. $E_{{{\text{PV}}}} { = }0$ for $v > v_{{{\text{limit}}}}$. When the impact radius approaches zero, $D{ = }0$.

Based on this impact scenario, the particle impact is laser-induced, the energy density of which follows Gaussian distribution. So, theoretically, the particle speed should also follow the Gaussian as well. Besides, the physical mechanism of impact and the actual shape of particles indicates a nearly symmetrical distribution as indicated by Fig. 1. The velocity distribution function is defined as a Gaussian function according to the experimental observation,

3 $$v\left( x \right) = v_{\max } \cdot e^{{ - x^{2} /2}} ,$$

where $v_{\max }$ denotes the peak velocity of the Gaussian velocity distribution. The threshold velocity $v_{{\text{T}}}$ ${v}_{T}$, for the PV cells to generate damage is

4 $$v_{T} = v_{\max } \cdot e^{{ - r^{2} /2}} ,$$

where $r$ represents the radius of the impact-induced damaged area, and

5 $$r = \sqrt {2\ln \left( {v_{\max } /v_{T} } \right)} .$$

The damaged area can be written as

6 $$S_{{{\text{damaged}}}} = \pi r^{2} = 2\pi \ln \left( {v_{\max } /v_{{\text{T}}} } \right),$$

here $$0<{v}_{T}\le {v}_{max}$$.

Thus, the damaged degree $D$ can be rewritten as

7 $$\begin{aligned} D & = \left( {\alpha \eta /S_{{{\text{damaged}}}} } \right)\iint\limits_{{S_{{{\text{damaged}}}} }} {\left[ {\rho_{{\text{p}}} \left( {v^{2} - v_{{\text{T}}}^{2} } \right)/\sigma_{{\text{s}}} } \right]dS} \\ & = \left[ {\alpha \eta \rho_{{\text{p}}} /\left( {\sigma_{{\text{s}}} S_{{{\text{damaged}}}} } \right)} \right]\iint\limits_{{S_{{{\text{damaged}}}} }} {\left( {v^{2} - v_{{\text{T}}}^{2} } \right)}dS \\ & { = }\left[ {\alpha \eta \rho_{{\text{p}}} /\left( {\sigma_{{\text{s}}} S_{{{\text{damaged}}}} } \right)} \right]\int\limits_{0}^{r} {\left( {v_{{{\text{max}}}}^{2} \cdot e^{{ - x^{2} }} - v_{{\text{T}}}^{2} } \right)} \cdot 2\pi xdx \\ & = \left[ {\alpha \eta \rho_{{\text{p}}} \pi v_{{{\text{max}}}}^{2} /\left( {\sigma_{{\text{s}}} S_{{{\text{damaged}}}} } \right)} \right]\left( {1 - e^{{ - r^{2} }} } \right) - \left[ {\left( {\alpha \eta \rho_{{\text{p}}} } \right)/\sigma_{{\text{s}}} } \right]v_{{\text{T}}}^{{2}} \\ & = \left[ {\alpha \eta \rho_{{\text{p}}} \pi \left( {v_{{{\text{max}}}}^{2} - v_{{\text{T}}}^{2} } \right)} \right]/\left( {\sigma_{{\text{s}}} S_{{{\text{damaged}}}} } \right) - \left[ {\left( {\alpha \eta \rho_{{\text{p}}} } \right)/\sigma_{{\text{s}}} } \right]v_{{\text{T}}}^{2} . \\ \end{aligned}$$

With Eq. (7), we obtain

8 $$E_{{{\text{PV}}}} = E_{{{\text{PV}}_{{0}} }} \cdot \left[ {1 - \left( {\alpha \eta \rho_{{\text{p}}} \pi \left( {v_{{{\text{max}}}}^{2} - v_{{\text{T}}}^{2} } \right)} \right)/\left( {\sigma_{{\text{s}}} S_{{{\text{all}}}} } \right) + \left( {\left( {\alpha \eta \rho_{{\text{p}}} } \right)/\left( {\sigma_{{\text{s}}} S_{{{\text{all}}}} } \right)} \right)v_{{\text{T}}}^{2} \cdot 2\pi \ln \left( {v_{\max } /v_{{\text{T}}} } \right)} \right].$$

Equation (8) can be rewritten as

9 $$E_{{{\text{PV}}}} = E_{{{\text{PV}}_{{0}} }} \cdot \left[ {1 - A \cdot \left( {v_{{{\text{max}}}}^{2} - B^{2} } \right) + 2AB^{2} \cdot \ln \left( {v_{\max } /B} \right)} \right].$$

where $$A = {{\alpha \eta \rho_{{\text{p}}} \pi } \mathord{\left/ {\vphantom {{\alpha \eta \rho_{{\text{p}}} \pi } {\sigma_{{\text{s}}} S_{{{\text{all}}}} }}} \right. \kern-\nulldelimiterspace} {\sigma_{{\text{s}}} S_{{{\text{all}}}} }}$$, $$B = v_{{\text{T}}}$$.

Therefore,

10 $$E_{{{\text{PV}}}} = \left\{ \begin{gathered} 1, \, v \le v_{{\text{T}}} \hfill \\ E_{{{\text{PV}}_{{0}} }} \cdot \left[ {1 - A \cdot \left( {v_{{{\text{max}}}}^{2} - B^{2} } \right) + 2AB^{2} \cdot \ln \left( {v_{\max } /B} \right)} \right] \hfill \\ 0, \, v \ge v_{{{\text{limit}}}} \hfill \\ \end{gathered} \right., \, v_{{\text{T}}} < v < v_{{{\text{limit}}}} .$$

By fitting the experimental data as shown in Fig. 4a based on Eq. (10), we obtain $A = 4.8 \times 10^{ - 5} \,{\text{s}}^{2} /{\text{m}}^{2}$ and $B = 89.6\,{\text{ m}}/{\text{s}}$, indicating that the threshold velocity $v_{{\text{T}}}$ is about 89.6 m/s for the PV cells to generate damage. Below this impact velocity, the efficiency reduction of the PV cell is negligible. Besides, the limiting velocity $v_{{{\text{limit}}}}$ can be determined in a range of 190 ~ 205 m/s according to the fitting results as shown in Fig. 4a. Once the impact velocity higher than $v_{{{\text{limit}}}}$, the PV cells will fail completely. Between $v_{{\text{T}}}$ and $v_{{{\text{limit}}}}$, the efficiency of the PV cells decreases quickly with higher impact velocity. The threshold velocity $v_{{\text{T}}}$, the limiting velocity $v_{{{\text{limit}}}}$, and the efficiency reduction obtained from the analysis model are consistent with the experimental results, which validate the proposed model for predicting the massive particles impact-induced conversion efficiency reduction, providing an effective method for performance degradation prediction of PV cells under various massive micro-particles impact conditions.

### Characterization of the PV cells after impact

**Figure 5:**
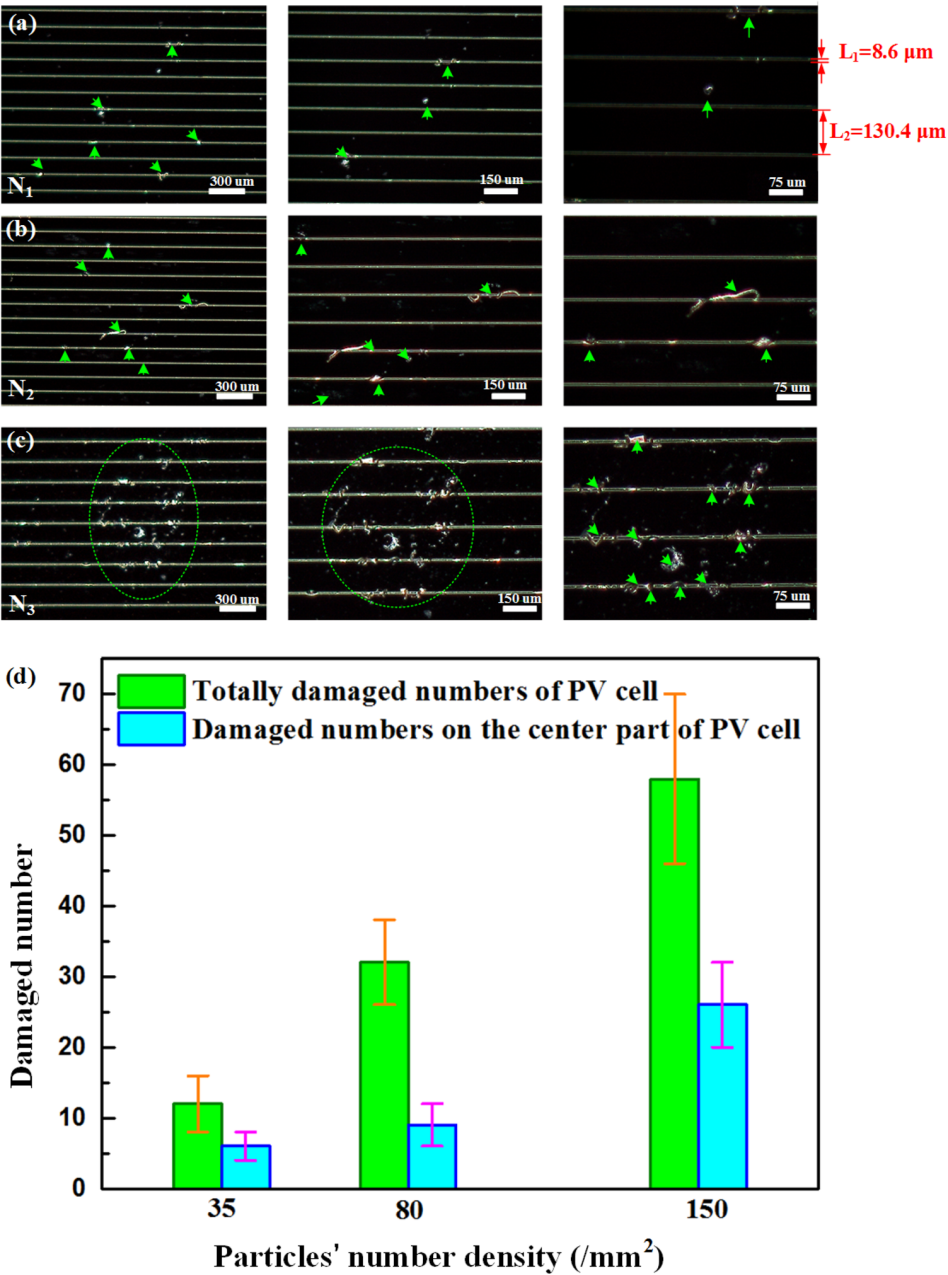
Three damaged patterns of the PV cells impacted by particles with (**a**) low, (**b**) medium, and (**c**) high particles number densities. (**d**) Relationship between damaged number and impact particles’ number density.

The damage morphologies for the three cases with different particle number densities are characterized by OM, as shown in Fig. 5. The results indicate that impact with high particles number density leads to severe damage in the PV cells as shown in Fig. 5a–c, where the green arrows indicate the apparent damages. The number of impact-induced damage regions in the whole PV cell and the center is counted and presented in Fig. 5d, clearly showing that the more severe damage is introduced by denser particle impact.

**Figure 6:**
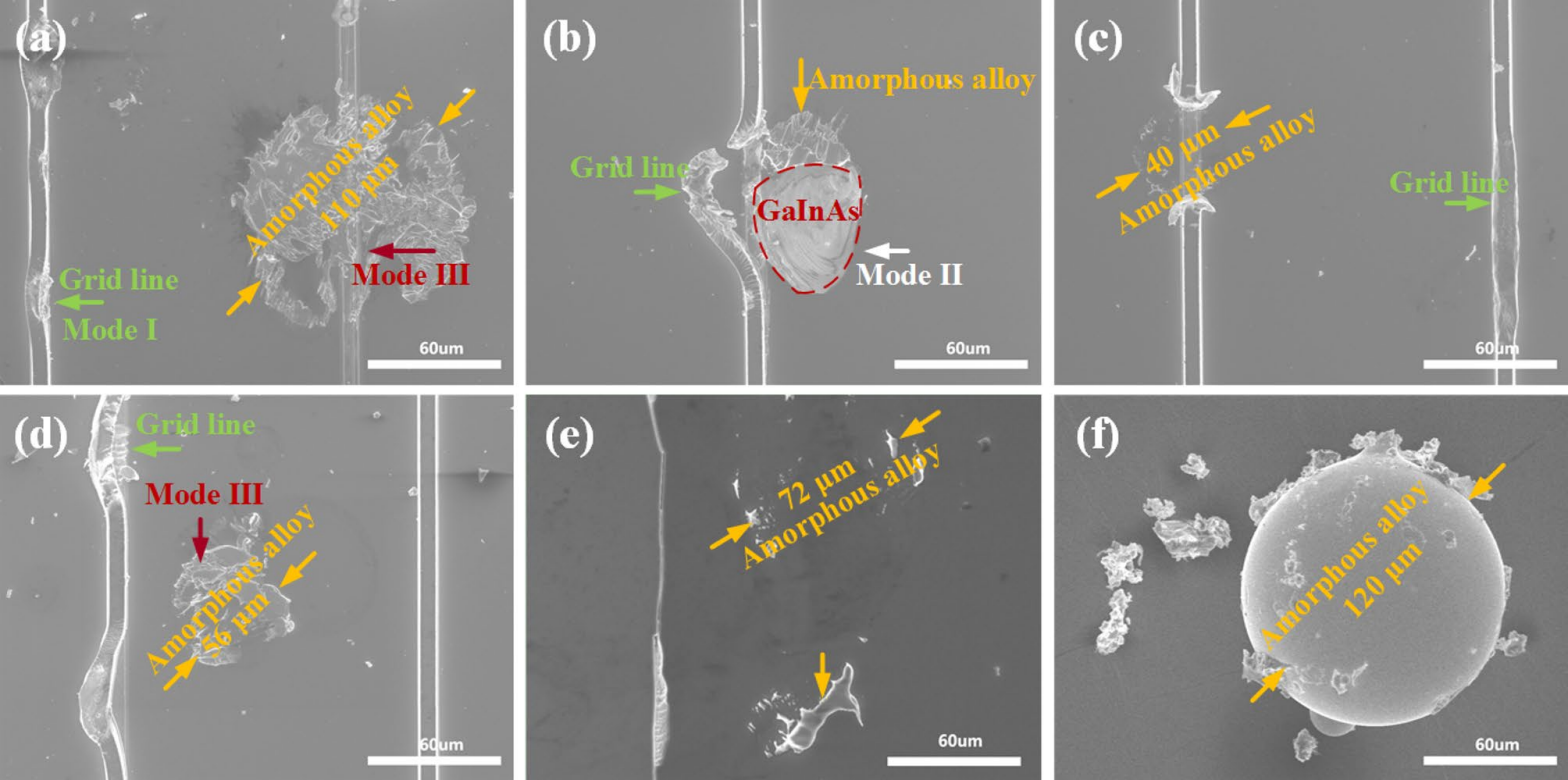
SEM images. (**a**) The residual particles and damage modes I and III. (**b**) The damaged PV cell sample and damage mode II. (**c–e**) The residual particles with different size after impact. (**f**) The original particle.

The typical damage patterns of the PV cell samples after impact are characterized by SEM as shown in Fig. 6. Figure 6a shows the impact indentation with a few particles left after impact, and most of the particles drop out of view. The diameters of the residues of the particles as depicted by the arrows are approximately 110 μm, which is slightly smaller than the original diameter of the particles. Besides, the grid line on the PV cell sample is also damaged as depicted by the green arrow in Fig. 6a. Figure 6b shows the broken of the grid lines made by Au after impact, which can definitely reduce the carrier collection ability and therefore decrease the efficiency of the PV cells. It is noteworthy that the inner GaInAs junction has been exposed to air after being impacted, indicating that the top GaInP junction is peeled-off and the structure of the triple-junction (GaInP/GaInAs/Ge) cell is destroyed. The residual particles can also be found in Fig. 6c–e with diameters of 40 μm, 56 μm, and 72 μm. Among the tested samples, all the residues are smaller than the size of the original particles of 120 ± 40 μm as measured in Fig. 6f, indicating that the particles are not fully melted during the impact.

**Figure 7:**
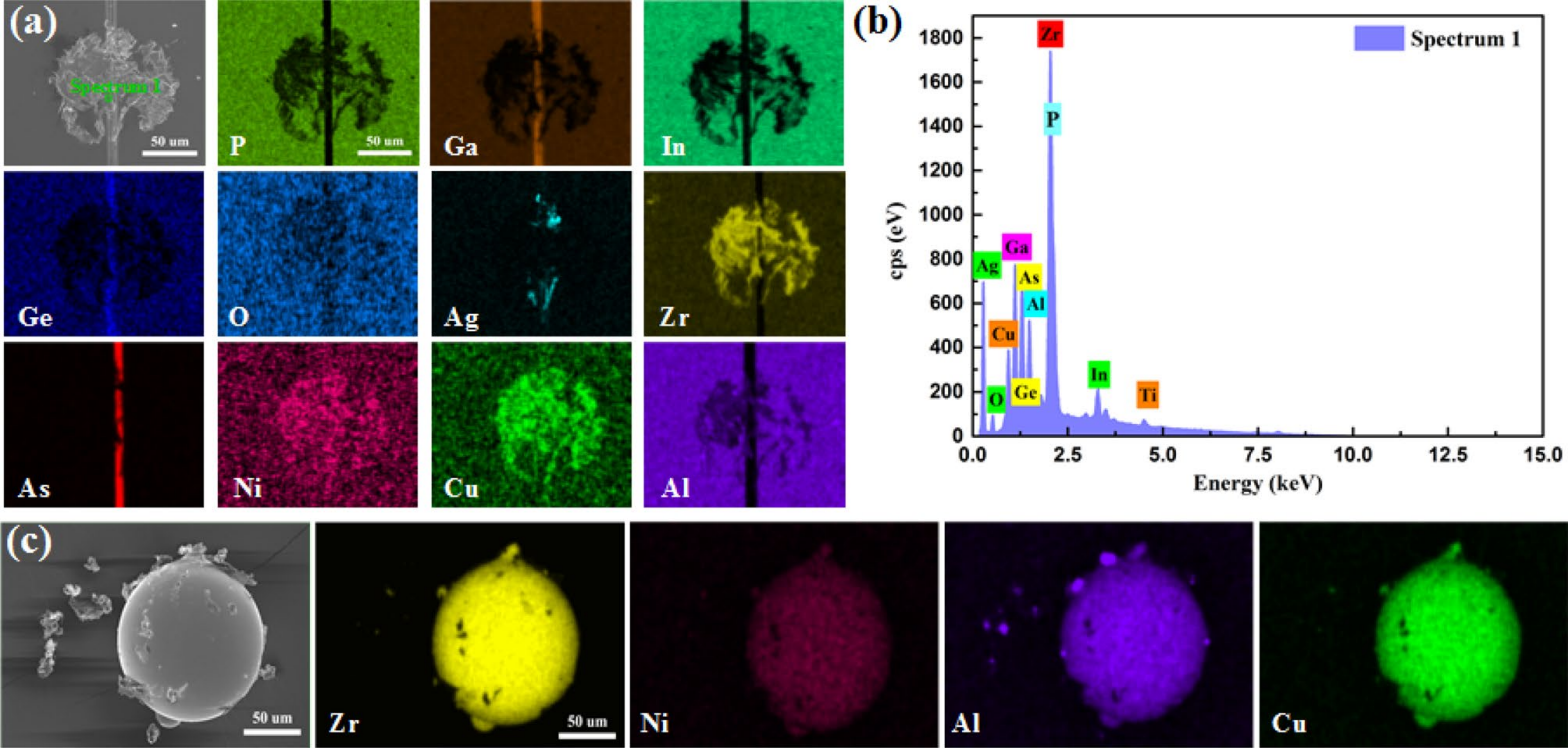
EDS mapping results. (**a**) PV cell after impact. (**b**) Distribution of elements in the PV cell sample after impact. (**c**) Distribution of elements in original amorphous alloy particles.

Furthermore, the element composition of the residual particles is recognized by EDS, operating at a primary voltage of 10 kV. Figure 7 shows the elements of the PV cells after the impact and the original particles. Figure 7a,b show the elements (e.g. Zr, Cu, Ni, and Al) of the amorphous alloy, which has been left on the PV cell samples after impact. In addition, the elements of the PV cell are also mapped. It shows that the particles damage the epilayer of the PV cell and the GaInAs layer is exposed to air, which is consistent with the results as given in Fig. 7b. Figure 7c shows the elements of the original amorphous alloy particles, i.e. Zr, Cu, Ni, and Al. The chemical elements of the particles used in the impact experiment are simpler when compared to the real dust particles, which have more complicated elements like earth's crust element (e.g. Al, Ma, Ca, and K) and metals elements (e.g. Zn, Mn, Ba, Cu, Ni, and Cr). However, as an impact event, it is reasonable to assume that the basic damage characteristics of the PV cell impacted by the particles in experiments can be extrapolated to the cases of real dust impact conditions in the natural environment.

### The damage modes in the PV cells

The performance degradation of the PV cells should be ascribed to the impact-induced damages. Therefore, it is crucial to characterize the mechanical damages due to particles’ impact on the PV cells to further reveal the mechanisms of performance degradation. The damage behavior of the PV cells in such an impact environment can be classified into three modes. The first damage mode, denoted by Mode I, is the rupture of the conducting grid lines by impact loadings as depicted by the green arrows in Fig. 6. The critical stress ($\sigma_{{\text{I}}}$) for the damage of the grid lines can be determined by the strength of Au, the material of the conducting lines. According to the test results in the literature^29^, regarding the material Au as elastic-perfectly plastic material, the strength of Au is

11 $$\sigma_{{{\text{Au}}}} = \sigma_{{\text{I}}} = 120 \, \,{\text{MPa}}.$$

If assuming the particles as a liquid during impact^30^, the threshold velocity, $v_{{\text{T}}}$, for the failure Mode I can be estimated as

12 $$\rho_{{\text{p}}} v_{{\text{T}}}^{2} /2 = \sigma_{{{\text{Au}}}} .$$

As a result, the estimated $v_{{\text{T}}}$ for the failure Mode I is about 111 m/s, which is close to the experimental observation, and the modeling results, indicating that the failure process of the PV cell might be initiated with the failure Mode I.

The second damage mode, denoted by Mode ΙΙ, corresponds to the damage of the PV cells as depicted by the white arrow in Fig. 6b. The damaged stress ($\sigma_{{{\text{II}}}}$) of the PV cells can be estimated by the classical elastic stress wave theory. For brittle materials such as the PV cells, the highest tensile stress dominates the fracture behavior of materials, which is the main damage mechanism. As a result, the maximum tensile stress criterion is applied for analyzing the fracture process of the material. The homogeneity is assumed for the PV cells to simplify the problem. According to the theory of one-dimensional stress wave^31^, a compressive stress pulse is generated under impact and propagated towards the PV cells. The compressive stress and the hoop stress arising within the PV cells at instantaneous contact approximately are^32^

13 $$\left. \sigma \right|_{t = 0} = \sigma_{{{\text{II}}}} = - v_{{\text{i}}} \cdot \left[ {\rho_{{\text{p}}} c_{{\text{p}}} \rho_{{\text{c}}} c_{{\text{c}}} /\left( {\rho_{{\text{p}}} c_{{\text{p}}} + \rho_{{\text{c}}} c_{{\text{c}}} } \right)} \right],$$

14 $$\sigma_{\varphi } = - \upsilon_{{\text{c}}} \sigma_{{{\text{II}}}} ,$$

where $\rho_{{\text{p}}}$ and $\rho_{{\text{c}}}$ denote the density of the particles and the PV cell, respectively. $v_{{\text{i}}}$ is the impact velocity of the particles and $\upsilon_{{\text{c}}}$ is Poisson's ratio of the PV cell. $c_{{\text{p}}}$ and $c_{{\text{c}}}$ are longitudinal wave velocities of the particles and the PV cell, respectively, which can be calculated as follow,

15a $$c_{{\text{p}}} = \sqrt {E_{{\text{p}}} /\rho_{{\text{p}}} } \cong 4130\,{\text{m}}/{\text{s}},$$

15b $$c_{{\text{c}}} = \sqrt {E_{{\text{c}}} /\rho_{{\text{c}}} } \cong 3090\,{\text{m}}/{\text{s}},$$

where $E_{{\text{p}}}$ and $E_{{\text{c}}}$ are elastic modulus of the particles and the PV cells, respectively.

Here, $v_{{\text{i}}} = 40\,{\text{m}}/{\text{s}}$ and $v_{{\text{i}}} = 171.3\,{\text{m}}/{\text{s}}$ at $t = 28$ μs are adopted in the calculation according to the high-speed camera results. The Poisson's ratio $\upsilon_{{\text{c}}} = C_{12} /\left( {C_{11} + C_{12} } \right) = 0.3297$, the elastic constants $C_{11} = 1.22 \times 10^{5} \,{\text{MPa}}$ and $C_{12} = 0.60 \times 10^{5} \,{\text{MPa}}$ are used in the analysis by linearly interpolation from the values for InP and GaP^9^. Therefore, the damaged stress $\sigma_{{{\text{II}}}}$, and the tensile hoop stress $\sigma_{\varphi }$, across the impact region for Mode ΙΙ could be determined as 0.41 GPa and 0.14 GPa for $v_{{\text{i}}} = 40\,{\text{m}}/{\text{s}}$, 1.74 GPa and 0.57 GPa for $v_{{\text{i}}} = 171.3\,{\text{m}}/{\text{s}}$, respectively, during the impact.

The third damage mode, denoted by Mode III, represents the bending effects indicated by the red arrow in Fig. 6a,d. The bending stress $\sigma_{{{\text{III}}}}$ in the PV cell could be calculated by the bending theory of clamped beams under a concentrated force $F$^33^, as determined by $F = p\pi r_{{\text{p}}}^{2}$, where $p = \rho_{{\text{p}}} v_{{\text{i}}}^{2} /2$ (determined as 4.45 MPa for $v_{{\text{i}}} = 40\,{\text{m}}/{\text{s}}$ and 81.58 MPa for $v_{{\text{i}}} = 171.3\,{\text{m}}/{\text{s}}$) is the dynamic pressure during impact, and *r* is the radius of the distributed pressure that is considered as equal to the radius of the particles,

16 $$\sigma_{{{\text{max}}}} = 4pr_{{\text{p}}}^{2} \left( {3 - 2r_{{\text{p}}} /R} \right)/\left( {6H_{{{\text{PV}}}}^{2} } \right),$$

where $r_{{\text{p}}} = 60$ μm and $R = 5.08\,{\text{mm}}$ are the radius of the particle and the equivalent radius of the PV cell, respectively. Substituting these values into Eq. (16) would yield $F = 0.05 \, N$, $\sigma_{{{\text{III}}}} = 2.13{\text{ MPa}}$ for $v_{{\text{i}}} = 40\,{\text{m}}/{\text{s}}$, and $F = 0.92 \, N$, $\sigma_{{{\text{III}}}} = \sigma_{{{\text{max}}}} = 40.47{\text{ MPa}}$ for $v_{{\text{i}}} = 171.3\,{\text{m}}/{\text{s}}$, respectively. It should be noted that when a cluster of particles impacts the PV cell, these values should be linearly superposed to obtain the realistic bending behavior of the PV cells.

The bending effect with no obvious cracks but residual particles on the PV cells results in the bending stresses and the residual stresses in the PV cells. Lee et al*.*^34^ investigated the effect of mechanical load on the PV modules by simulation and the results indicated the cell near the center experienced residual tensile stresses with the highest value of 207.6 MPa under uniformly distributed loading with an amplitude of 5.4 kPa. Ojo and Paggi^10^ proposed a novel 3D coupled thermo-visco-elastic shear-lag model to determine the stress distribution in the PV modules after lamination. The results showed the compressive stresses with amplitudes of 40 ~ 65 MPa and 140 MPa were introduced along the edges of the Silicon cells and in the mid-portion of the laminate, respectively. The bending stress in the PV cells of the present study is comparable to that obtained by Lee et al*.*^34^ and Ojo and Paggi^10^.

It is noteworthy that the aforementioned three damage modes in the PV cells will affect the light-electricity conversion efficiency and other electrical performance through different mechanisms. Those effects are generally mixed as indicated by the I–V curves in Fig. 3. In brief, the breakage of the grid lines (Mode I) will decrease the carrier collection efficiency due to a reduction in effective conductance. The fracturing of the cell material (Mode II) will decrease the performing area of the PV cell, and the residual stresses resulted from all of the failure modes (Mode I, Mode II, and Mode III) in the PV cells will influence the energy band in a rather complex way and change the overall performance of the PV cells. In addition, the local impact-induced dents on the surface of the PV cells can also change the light irradiation angles and therefore decrease the conversion efficiency. In the future, we will try to investigate the individual effects of each damage mode on the light-electricity conversion performance. The single impact event of the cluster particles with high velocity should be able to somewhat reflect the damage accumulation effects of the PV cells under long-term but relatively low-velocity impacts in the service environment. The scaling law on the degradation effect of the PV cells between the laser-induced short-time high-velocity impact and the long-term low-velocity impact in the service environment exposure will be investigated in the future. In addition, we will engage in studying more types of PV cells in the future for the reason that the current study is focused on a particular type of PV cells.

## Conclusions

In this paper, the mechanical damage modes and the degradation behavior of PV cells subjected to massive particles impact with high velocity are studied by experimental and modeling approaches. The main conclusions can be summarized as follows.

1. A well-controlled laser-driven micro-particles impact experimental method is built with particle diameters ranging from several to hundreds of micrometers and impact velocities ranging from tens to hundreds of m/s for PV cells, providing a practical method to investigate the impact-induced performance degradation of PV cells in the laboratory.
2. The performance degradation behavior of the PV cells under various impact velocities is observed. The conversion efficiency sharply decreases with impact velocity increasing. The critical impact velocities for the initiation and total failure of the PV cells are determined.
3. A physical model based on massive impact-induced damage mechanisms is developed and validated by experiments, providing an effective method for performance degradation prediction of PV cells under various massive micro-particles impact conditions.
4. Three damage modes are observed including damaged conducting grid lines, fractured PV cell surfaces, and the bending effects after impact. The corresponding strength of each model is comprehensively quantified by mechanical theory.

In the future, the experimental method will be improved to discern the effects of individual failure modes on the PV cells. In addition, the scaling law based on the conservation of kinetic energy will be established to bridge the gap between the short-time high-velocity impact in the laboratory and the long-term low-velocity impact in the field.

## Material and methods

### PV cells

**Figure 8:**
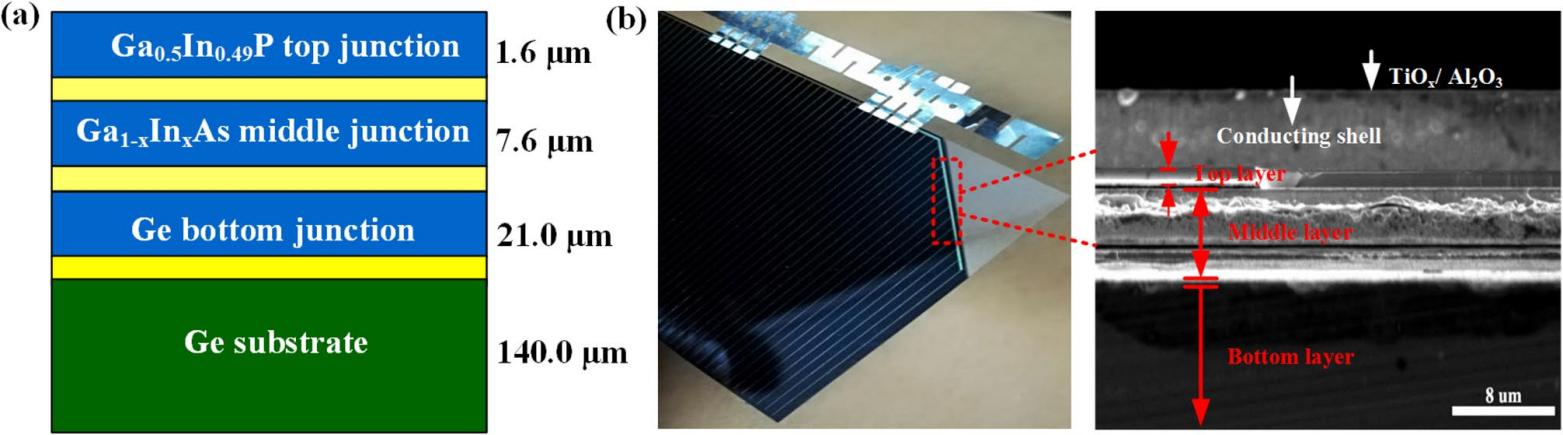
(**a**) Structure sketch of the PV cell used in experiments^35^. (**b**) Optical photo and cross-sectional SEM image of the triple-junction GaInP/GaInAs/Ge thin film.

The structure of the triple-junction thin-film PV cells (GaInP/GaInAs/Ge) with Ge substrates used in experiments (Fullsuns Company in Shanghai, China) is shown in Fig. 8a, where only the primary layers are depicted. Figure 8b shows the optical photo of a sample cell and its cross-sectional SEM image taken from the edge of the PV cell to exclude cutting damage. The in-plane dimension of the PV cell sample is $9.0 \times 9.0$ mm^2^ and its thickness is 170.2 μm. The effective density (*ρ*_c_) of the PV cell is 5.88 g/cm^3^. The conducting line is made of gold and the antireflection film of TiO_x_/Al_2_O_3_ on the top of the cell is covered by a glass layer (KFB120) with a thickness of 120 ± 20 μm. A more detailed configuration of the PV cell can be found in Ref.^35^. It is to be noted that the stability of the PV cells used in the present work is further enhanced through improving the lattice match^3^.

### Experimental method

**Figure 9:**
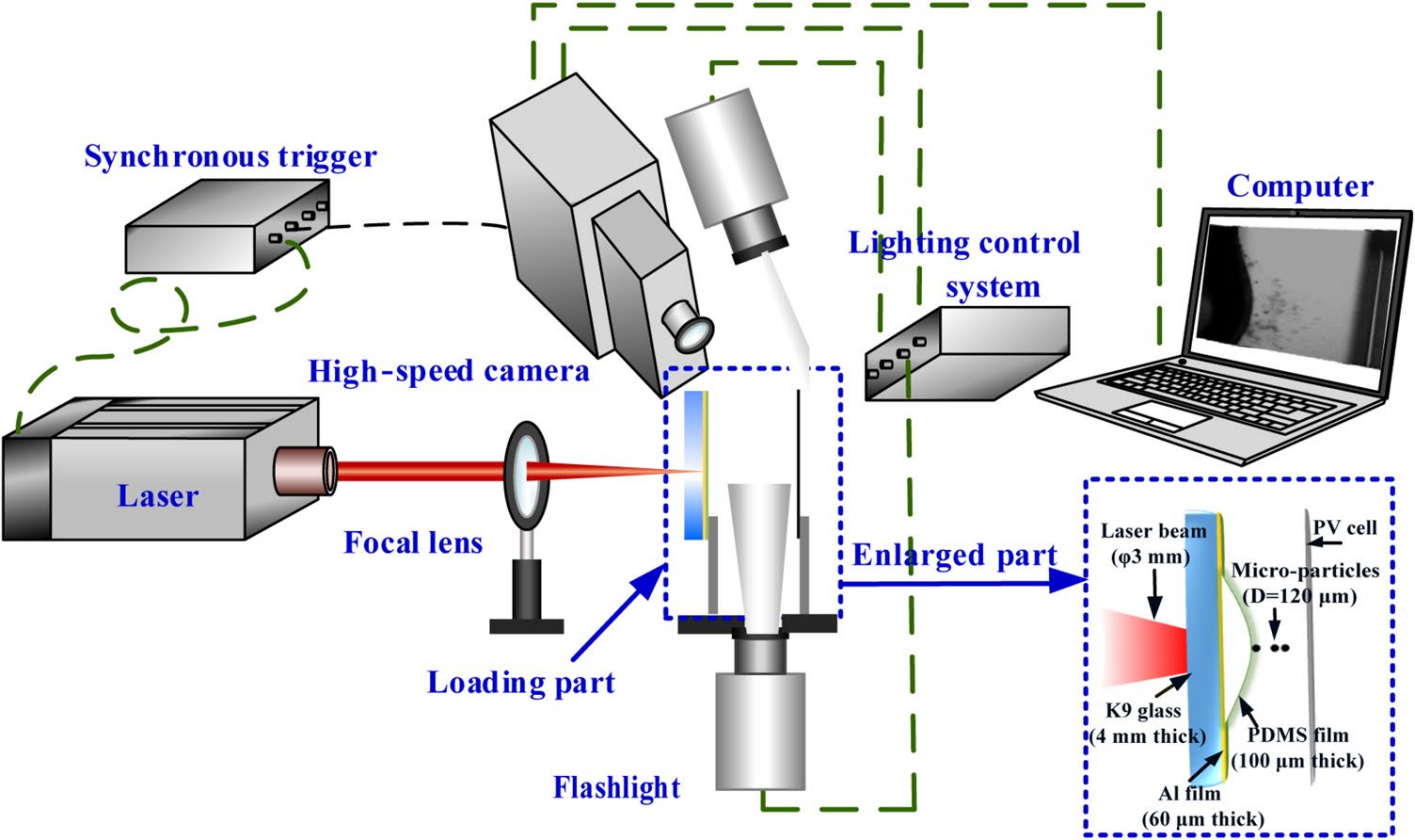
Schematic of the laser-shock induced particles impact on PV cells.

The method of laser-driven particles impact is developed to simulate the environmental impact response of the PV cells. The schematic of the impact experiment is shown in Fig. 9. The laser-shock-driven impact is performed with a Q-switch high power Nd: YAG pulsed laser, operating at a wavelength of 1064 nm and 2.5 J output energy per shot. The temporal profile of the laser power density is nearly in Gaussian distribution with a full width at half maximum (FWHM) approximately 10 ns^36^. The top-head spatial distribution of the laser power density is modulated to be nearly uniform^37,38^. The initial diameter of the laser beam is 16 mm, which is focused on the aluminum foil by a lens with a focal length of 600 mm. The diameter of the focused laser beam is about 3 mm.

A cluster of amorphous alloy particles (Zr_55_Cu_30_Ni_5_Al_10_, the density *ρ*_p_ = 5.56 g/cm^3^, the elastic modulus *E*_p_ = 95 GPa, diameter *d* = 120 ± 40 μm) is propelled through fast-expanding plasma generated by the laser ablation on the surface of a 60-μm-thick aluminum foil. The diameter of particles adopted in the experiments is corresponding to the scale of real dust (i.e. smaller than 500 µm). Meanwhile, a 100-μm-thick polydimethylsiloxane (PDMS) layer is used to confine the ablation products, eliminate the temperature effect, and attenuate the shock waves to further accelerate the particles. A 4-mm-thick overlay of K9 glass is fully-clamped without a cushion at the back surface by a designed holder against the laser irradiation. The high-power density laser, the high-speed camera (specialised-imaging SIMD16) with 5 × 10^5^ fps, and two high power flashlights are triggered synchronously to capture the impact process of the particles towards the PV cells. Additionally, the distance between the PV cell and the initial position of the particles is about 10 mm to avoid the following impact of PDMS film as depicted in Fig. 9.

### Light-electricity conversion test

The I–V curves of solar cells are obtained by a Keithley 2400 source meter (Keithley, USA) under the illumination of AM 1.5, 100 mW/cm^2^ by an Ivtest Station 4000AAA solar simulator (CrownTech, USA). In this case, the height of the lamp is adjusted to cover the whole surface of the PV cells, which is calibrated by a standard luminometer to maintain the light intensity upon the specimen as 100 mW/cm^2^. I–V characterizer test performs a voltage sweep ranging from 0 to 2600 mV at a current limit of 7 mA. It is worth mentioning that the PV cells are selected as small size for simplicity and practical use in the indoor experiment. The electrical specification of the PV cells used in this work is given in Table 2. It is worth mentioning that the PV cells are selected as small size for simplicity and practical use in the indoor experiment.

**Table 2:** Electrical specifications of the PV cells used in this work.

Parameters	Values
Type	TRIPLE-junction GaInP/GaInAs/Ge
Output tolerance	0 ~ ± 5%
Maximum voltage	2.43 V
Maximum current	0.014 A
Maximum power, *P*_max_	0.034 W
Open circuit voltage, *V*_oc_	2.63 V
Short circuit current, *I*_sc_	0.0144 A
Test conditions	100 mW/cm^2^, AM 1.5 T = 25 ℃

## Supplementary Information

Supplementary information.

## Data Availability

The datasets generated and analysed during the current study are available from the corresponding author on reasonable request.

## References

[1] Posthuma NE, Heide JVD, Flamand G, Poortmans J (2007). Emitter formation and contact realization by diffusion for germanium photovoltaic devices. IEEE Trans. Electron Dev..

[2] Lin TW, Rowe LP, Kaczkowski AJ, Horn GP, Johnson HT (2016). Polarized light emission from grain boundaries in photovoltaic silicon. Extreme Mech. Lett..

[3] King RR (2007). 40% efficient metamorphic GaInP∕GaInAs∕Ge multijunction solar cells. Appl. Phys. Lett..

[4] Chen Z (2018). Random forest based intelligent fault diagnosis for PV arrays using array voltage and string currents. Energy Convers. Manag..

[5] Vidal K, de Oliveira A, Aghaei M, Rüther R (2020). Aerial infrared thermography for low-cost and fast fault detection in utility-scale PV power plants. Sol. Energy..

[6] Liu Z, Lu Y, Kong J, Gong J, Wang S (2020). Multimodal fault-tolerant control for single-phase cascaded off-grid PV-storage system with PV failure using hybrid modulation. Microelectron. Reliab..

[7] Pei T, Zhang J, Li L, Hao X (2020). A fault locating method for PV arrays based on improved voltage sensor placement. Sol. Energ..

[8] Kaldellis JK, Kapsali M (2011). Simulating the dust effect on the energy performance of photovoltaic generators based on experimental measurements. Energy.

[9] Eggenhoffner, R. & Landolt-Börnstein. Numerical data and functional relationships in science and technology—Group III, Vol. 7, Part d2. (1982).

[10] Ojo SO, Paggi M (2016). A 3D coupled thermo-visco-elastic shear-lag formulation for the prediction of residual stresses in photovoltaic modules after lamination. Compos. Struct..

[11] Gomes ILR, Melicio R, Mendes VMF (2020). Dust effect impact on PV in an aggregation with wind and thermal powers. Sustain. Energy Grids.

[12] Figgis B, Ennaoui A, Ahzi S, Rémond Y (2017). Review of PV soiling particle mechanics in desert environments. Renew. Sustain. Energy. Rev..

[13] Hachicha AA, Al-Sawafta I, Said Z (2019). Impact of dust on the performance of solar photovoltaic (PV) systems under United Arab Emirates weather conditions. Renew. Energy..

[14] Pavan AM, Mellit A, Pieri DD, Kalogirou SA (2013). A comparison between BNN and regression polynomial methods for the evaluation of the effect of soiling in large scale photovoltaic plants. Appl. Energy..

[15] Javed W, Wubulikasimu Y, Figgis B, Guo B (2017). Characterization of dust accumulated on photovoltaic panels in Doha, Qatar. Sol. Energy..

[16] Chen J (2020). Study on impacts of dust accumulation and rainfall on PV power reduction in East China. Energy.

[17] Memiche M, Bouzian C, Benzahia A, Moussi A (2020). Effects of dust, soiling, aging, and weather conditions on photovoltaic system performances in a Saharan environment—Case study in Algeria. Glob. Energy. Interconnect..

[18] Gholami A, Khazaee I, Eslami S, Zandi M, Akrami E (2018). Experimental investigation of dust deposition effects on photo-voltaic output performance. Sol. Energy..

[19] Alnaser NW (2018). Comparison between performance of man-made and naturally cleaned PV panels in a middle of a desert. Renew. Sustain. Energy. Rev..

[20] Fraga MM, Campos BLDO, Almeida TBD, Fonseca JMFD, Lins VDFC (2018). Analysis of the soiling effect on the performance of photovoltaic modules on a soccer stadium in Minas Gerais. Brazil. Sol. Energy..

[21] Connolly D, Lund H, Mathiesen BV, Leahy M (2010). A review of computer tools for analysing the integration of renewable energy into various energy systems. Appl. Energy..

[22] Lee JH, Loya PE, Lou J, Thomas EL (2014). Dynamic mechanical behavior of multilayer graphene via supersonic projectile penetration. Science.

[23] Hassani-Gangaraj, M., Veysset, D., Nelson, K. A. & Schuh, C. A. Melting can hinder impact-induced adhesion. *Phys. Rev. Lett.* **119** (2017). 10.1103/PhysRevLett.119.175701 29219456

[24] Hassani-Gangaraj M, Veysset D, Nelson KA, Schuh CA (2018). Melt-driven erosion in microparticle impact. Nat. Commun..

[25] Alahmad M, Chaaban MA, Lau SK, Shi J, Neal J (2012). An adaptive utility interactive photovoltaic system based on a flexible switch matrix to optimize performance in real-time. Sol. Energy..

[26] Wendelin, T. In *Asme International Solar Energy Conference.*

[27] Kalogirou SA, Agathokleous R, Panayiotou G (2013). On-site PV characterization and the effect of soiling on their performance. Energy.

[28] Jun Z, Xing Z (1995). The asymptotic study of fatigue crack growth based on damage mechanics. Eng. Fract. Mech..

[29] Greer JR, Oliver WC, Nix WD (2005). Size dependence of mechanical properties of gold at the micron scale in the absence of strain gradients. Acta Mater..

[30] Torenbeek, E. & Wittenberg, H. In *Flight Physics: Essentials of Aeronautical Disciplines and Technology with Historical Notes* 87–123 (2009).

[31] Boedoni, P. G. Stress waves in solids. (1953).

[32] Wu ZL, Wu CW, Chen GN, Zhang K (2010). On a novel method of impact by a front-end-coated bullet to evaluate the interface adhesion between film and substrate. Prog. Org. Coat..

[33] Timoshenko, S. & Woinowsky-krieger, S. Theory of plates and shells. (1959).

[34] Yixian (2013). Stress analysis of silicon wafer-based photovoltaic modules under IEC 61215 mechanical load test. Energy Proc..

[35] Yuan YC, Wu CW (2015). Thermal analysis of film photovoltaic cell subjected to dual laser beam irradiation. Appl. Therm. Eng..

[36] Wu X, Yin Q, Huang C (2015). Experimental study on pressure, stress state, and temperature-dependent dynamic behavior of shear thickening fluid subjected to laser induced shock. J. Appl. Phys..

[37] Wu X, Zhong F, Yin Q, Huang C (2015). Dynamic response of shear thickening fluid under laser induced shock. Appl. Phys. Lett..

[38] Wu X (2011). Shock pressure induced by glass-confined laser shock peening: Experiments, modeling and simulation. J. Appl. Phys..

