# Supplementary material for: Study on performance degradation and damage modes of thin-film photovoltaic cell subjected to particle impact
Source: Sci Rep. 2021 Jan 12;11:782. doi: 10.1038/s41598-020-80879-w (PMC7804249; doi:10.1038/s41598-020-80879-w)
Supplement: Supplementary file 1 — Supplementary information. [file 41598_2020_80879_MOESM1_ESM.pdf]

Supporting Information

Study on performance degradation and damage modes of thin-film photovoltaic cell subjected to particle impact

Kailu Xiao[a, b], Xianqian Wu[a, *], Xuan Song[a, b], Jianhua Yuan[c], Wenyu Bai[d], Chenwu Wu[a, *] and Chenguang Huang[a]

a Institute of Mechanics, Chinese Academy of Sciences, Beijing, 100190, China

b School of Engineering Science, University of Chinese Academy of Sciences, Beijing, 100049, China

c College of Electrical Engineering & Renewable Energy, Three Gorges University, Yichang, 443002, China

d Department of Medical Engineering, California Institute of Technology, Pasadena, CA 91125, USA

[*] Corresponding authors. Institute of Mechanics, Chinese Academy of Sciences. No.15 Beisihuanxi Road, Haidian District, Beijing 100190, China. Email: wuxianqian@imech.ac.cn (X. Wu) & chenwuwu@imech.ac.cn (C. Wu).

1. Three fitted probability distributions and the MSE value

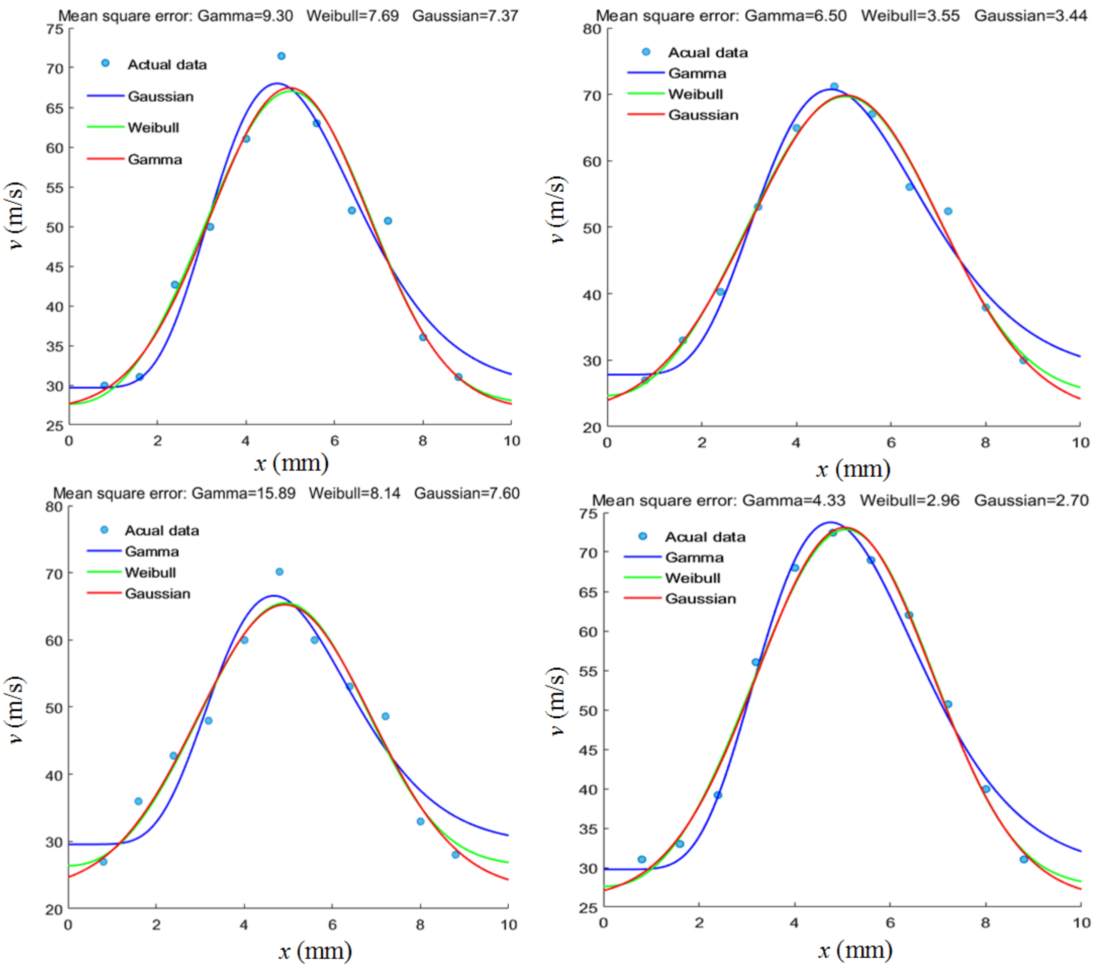

Fig. S1 Four sets of impact velocity data fitted by three probability distributions and their MSE values.
